# Evaluation of Pregnancy Outcomes Among Women With Decreased Fetal Movements

**DOI:** 10.1001/jamanetworkopen.2021.5071

**Published:** 2021-04-08

**Authors:** Jessica M. Turner, Vicki Flenady, David Ellwood, Michael Coory, Sailesh Kumar

**Affiliations:** 1 Mater Research, University of Queensland, South Brisbane, Queensland, Australia; 2 Faculty of Medicine, University of Medicine, South Brisbane, Queensland, Australia; 3 National Heath and Medical Research Council Centre of Research Excellence in Stillbirth, Mater Research Institute, University of Queensland, Brisbane, Queensland, Australia

## Abstract

**Importance:**

Stillbirth is a devastating pregnancy outcome with far-reaching economic and psychosocial consequences, but despite significant investment, a screening tool for identifying those fetuses at risk for stillbirth remains elusive. Maternal reporting of decreased fetal movements (DFM) has been found to be associated with stillbirth and other adverse perinatal outcomes.

**Objective:**

To examine pregnancy outcomes of women presenting with DFM in the third trimester at a tertiary Australian center with a clear clinical management algorithm.

**Design, Setting, and Participants:**

This cohort study used data on all births meeting the inclusion criteria from 2009 through 2019 at Mater Mothers’ Hospital in Brisbane, Australia. This is a tertiary center and Australia’s largest maternity hospital. All singleton births without a known congenital anomaly after 28 weeks’ gestation were included. Among 203 071 potential participants identified from the hospital database, 101 597 individuals met the eligibility criteria. Data analysis was performed from May through September 2020.

**Exposure:**

Presentation to hospital with DFM after 28 weeks gestation.

**Main Outcomes and Measures:**

The primary outcome of this study was the incidence of stillbirth. Multivariate analysis was undertaken to determine the association between DFM and stillbirth, obstetric intervention, and other adverse outcomes, including being born small for gestational age (SGA) and a composite adverse perinatal outcome (at least 1 of the following: neonatal intensive care unit admission, severe acidosis [ie, umbilical artery pH <7.0 or base excess −12.0 mmol/L or less], 5-minute Apgar score <4, or stillbirth or neonatal death). The hypothesis being tested was formulated prior to data collection.

**Results:**

Among 101 597 women with pregnancies that met the inclusion criteria, 8821 (8.7%) presented at least once with DFM and 92 776 women (91.3%) did not present with DFM (ie, the control population). Women presenting with DFM, compared with those presenting without DFM, were younger (mean [SD] age, 30.4 [5.4] years vs 31.5 [5.2] years; *P* < .001), more likely to be nulliparous (4845 women [54.9%] vs 42 210 women [45.5%]; *P* < .001) and have a previous stillbirth (189 women [2.1%] vs 1156 women [1.2%]; *P* < .001), and less likely to have a previous cesarean delivery (1199 women [13.6%] vs 17 444 women [18.8%]; *P* < .001). During the study period, the stillbirth rate was 2.0 per 1000 births after 28 weeks’ gestation. Presenting with DFM was not associated with higher odds of stillbirth (9 women [0.1%] vs 185 women [0.2%]; adjusted odds ratio [aOR], 0.54; 95% CI, 0.23-1.26, *P* = .16). However, presenting with DFM was associated with higher odds of a fetus being born SGA (aOR, 1.14; 95% CI, 1.03-1.27; *P* = .01) and the composite adverse perinatal outcome (aOR, 1.14; 95% CI, 1.02-1.27; *P* = .02). Presenting with DFM was also associated with higher odds of planned early term birth (aOR, 1.26; 95% CI, 1.15-1.38; *P* < .001), induction of labor (aOR, 1.63; 95% CI, 1.53-1.74; *P* < .001), and emergency cesarean delivery (aOR, 1.18; 95% CI, 1.09-1.28; *P* < .001).

**Conclusions and Relevance:**

The presence of DFM is a marker associated with increased risk for a fetus. This study’s findings of a nonsignificantly lower rate of stillbirth among women with DFM may be reflective of increased community awareness of timely presentation to their obstetric care clinician when concerned about fetal movements and the benefits of tertiary level care guided by a clear clinical management protocol. However, DFM was associated with increased odds of an infant being born SGA, obstetric intervention, early term birth, and a composite of adverse perinatal outcomes.

## Key Points

**Question:**

What are the pregnancy outcomes in women presenting with decreased fetal movements (DFM) in a tertiary center with a clear management algorithm?

**Findings:**

In this cohort study of more than 100 000 women, DFM was not associated with an increased risk of stillbirth. There was a significant association with the infant being born small for gestational age, planned early term birth, operative birth, and a composite of adverse perinatal outcomes.

**Meaning:**

Although DFM, as managed by a clinical algorithm, was not associated with an increased risk of stillbirth, it was associated with identification of a fetus at risk of a number of adverse outcomes, including being small for gestational age and iatrogenic early term birth.

## Teaser

This cohort study evaluates the association of decreased fetal movements with odds of stillbirth in an Australian tertiary care center.

## Introduction

Reducing the tragedy of the estimated 2 million deaths that occur in the antenatal and intrapartum period remains the focus of significant national and international^1^ efforts. In low-income and middle-income countries, almost 1 in 2 stillbirths occurs during labor. In high-income countries, most stillbirths occur in the antenatal period,^2^ thus potentially allowing time to mitigate this risk through lifestyle and behavior change, optimization of management of comorbidities, identification of fetuses who are small for gestational age (SGA),^3,4^ and education regarding the importance of monitoring fetal movements. However, the causative pathways that culminate in fetal death are often poorly understood, making complete prevention of stillbirth problematic. Because of this difficulty, most stillbirth mitigation strategies involve a package of interventions addressing different elements of prenatal care, education, and risk factors.^5,6^

Movements provide 1 simple measure of fetal well-being. Perception of fetal movements that are normal for that pregnancy generally reflects an appropriately functioning central nervous system and adequate oxygenation.^7^ Fetal hypoxia, associated with acute or chronic placental dysfunction, induces activation of the peripheral chemoreflex, centralization of cardiac output to vital organs, and a reduction in fetal movements, thereby limiting energy expenditure and oxygen consumption.^8,9^ Although decreased fetal movements (DFM) are associated with infants being born SGA, stillbirth, higher rates of induction of labor (IOL), emergency cesarean delivery, and adverse neonatal outcomes,^7,9,10,11,12,13,14,15,16^ the usefulness of DFM in predicting poor obstetric and perinatal outcomes is questionable, with most women who report DFM in the third trimester having outcomes without complications.^17^ Furthermore, maternal perception of fetal movements is highly subjective, and there is no universally agreed upon definition.^18^

Currently, many international guidelines emphasize DFM as an important warning sign associated with risk of stillbirth for the fetus, and women are urged to monitor their baby’s movements and alert their clinicians if concerned.^19,20,21,22,23^ However, the evidence supporting the incorporation of DFM into national guidelines and as part of a broader stillbirth reduction strategy remains limited.^5,14,22,24,25,26,27^ The aim of this study was to review pregnancy outcomes of women with singleton pregnancies presenting with DFM in the third trimester at a large Australian perinatal center.

## Methods

This was an 11-year (ie, 2009-2019) retrospective cohort study of women giving birth at the Mater Mothers’ Hospital in Brisbane, Australia. Ethical and governance approvals were obtained from the Mater Research Human Research Ethics Committee and Governance office, respectively (Ref No. HREC/18/MHS/46). A waiver of consent was granted for this study by the Mater Research Human Research Ethics Committee because of minimal risk to patients, sufficient protection of the participants’ privacy, and protection of confidential data (in keeping with the national statement).^28^ This study has been reported according to the Strengthening the Reporting of Observational Studies in Epidemiology (STROBE) reporting guideline and checklist.

Women with a single fetus without a known congenital anomaly presenting with DFM (ie, a decrease in frequency or strength of fetal movements, a complete absence in fetal movements, or a deviation from the previous pattern of movements as perceived by the woman) after 28 weeks and 0 days’ gestation were eligible for inclusion. Gestational age was calculated from the last menstrual period or from a first trimester ultrasonography scan. Women who presented with DFM but had a confirmed intrauterine fetal demise at first presentation were excluded from analysis.

At our institution, the management of DFM changed over the study period. From 2009 to 2016, all women who presented with DFM received electronic fetal heart rate (FHR) monitoring as an initial screen of fetal well-being. Additional investigations were performed at the discretion of the treating obstetric team. However, beginning in 2016, prior to a change in national recommendations, hospital policy was amended so that all women presenting with DFM after 28 weeks and 0 days’ gestation received additional measures beyond electronic FHR monitoring: a blood test to detect feto-maternal hemorrhage (ie, Kleihauer Betke test^29^) and consideration for ultrasonography scan to assess fetal growth and well-being^22^ (eFigure 1 in the Supplement).

The primary outcome of this study was the incidence of stillbirth. Secondary outcomes included rates of IOL, planned preterm birth (ie, planned cesarean delivery or IOL <37 weeks and 0 days’ gestation), planned early term birth (ie, planned cesarean delivery or IOL between 37 weeks and 0 days’ and 38 weeks and 6 days’ gestation), vaginal birth, emergency cesarean delivery, an infant born SGA (ie, birth weight <10th centile for gestational age and sex^30^), and a composite of severe perinatal outcomes (ie, neonatal intensive care unit [NICU] admission, severe acidosis [ie, umbilical artery pH <7.0 or base excess −12.0 mmol/L or less], 5-minute Apgar score <4, or stillbirth or neonatal death). Additional intrapartum and neonatal outcomes included mode of birth, pathological intrapartum FHR patterns (as determined by the treating obstetric team, based on guidelines from the Royal Australian and New Zealand College of Obstetricians and Gynaecologists^31^), meconium stained liquor, gestational age at birth, birth weight, 5-minute Apgar score greater than 7 and less than 4, severe acidosis, respiratory distress, NICU admission, and neonatal death within 28 days of birth.

### Statistical Analysis

Data were reported as mean (SD) or median (interquartile range [IQR]), and associations between variables were assessed using Pearson χ^2^, *t* test, or Wilcoxon rank sum. Logistic regressions were presented as odds ratios (ORs) or adjusted ORs (aORs) with 95% CIs. Multiple logistic regression was undertaken, with adjustment for IOL, elective cesarean delivery, mode of birth, gestational age at birth, birth weight, and year of birth when appropriate.

A subgroup analysis was performed to assess outcomes associated with the change in practice after the incorporation of national DFM guidelines into hospital policy in 2016. Subgroup analyses were also undertaken to ascertain the association of the number of DFM presentations and gestational age at first presentation with clinical outcomes. Statistical significance was determined by *P* ≤ .05, and *P* values were 2-sided. Statistical analyses were performed from May through September 2020 using Stata/SE statistical software version 15 (StataCorp).

## Results

Among 101 597 women over the 11-year study period, 8821 women (8.7%) presented with DFM at a median (IQR) gestational age of 37.0 (34.0-38.5) weeks, and 92 776 women (91.3%) did not present with DFM (Figure). Women with DFM, compared with women without DFM, were significantly younger (mean [SD] age, 30.4 [5.4] years vs 31.5 [5.2] years; *P* < .001), had a higher median (IQR) body mass index (calculated as weight in kilograms divided by height in meters squared; 24.3 [21.4-28.4] vs 23.0 [20.7-26.6]; *P* < .001), and were more likely to be nulliparous (4845 women [54.9%] vs 42 210 women [45.5%]; *P* < .001). They were less likely to be White individuals (5356 women [60.7%] vs 61 705 women [66.5%]; *P* < .001) and less likely to have had a previous cesarean delivery (1199 women [13.6%] vs 17 444 women [18.8%]; *P* < .001) or smoke (898 women [10.2%] vs 11 104 women [12.0%]; *P* < .001). (Table 1) Women with DFM were more likely to have had a previous stillbirth (189 women [2.1%] vs 1156 women [1.2%]; *P* < .001) or have diabetes (994 women [14.0%] vs 8475 women [10.0%]; *P* < .001). Among women with DFM, 7487 women (84.9%) presented with DFM once, while 1334 women (15.1%) presented twice or more. We excluded 33 women from the final analyses because an intrauterine fetal demise was diagnosed at first presentation.

**Figure.**
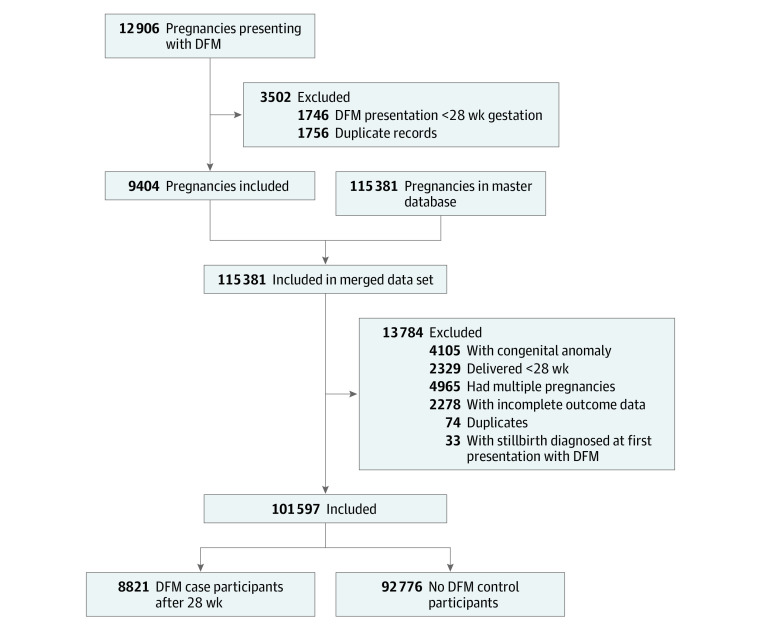
Study Population Diagram DFM indicates decreased fetal movements.

**Table 1.** Maternal Demographic Characteristics

Characteristic	No. (%)		*P* value
	With DFM (n = 8821)	Without DFM (n = 92 776)	
Maternal age, mean (SD)	30.4 (5.4)	31.5 (5.2)	<.001
Maternal BMI, median (IQR)	24.3 (21.4-28.4)	23.0 (20.7-26.6)	<.001
Race/ethnicity			
White	5356 (60.7)	61 705 (66.5)	<.001
Indigenous	217 (2.5)	1817 (2.0)	.001
Asian	1698 (19.2)	16 342 (17.6)	<.001
Other ethnicity	966 (11.0)	9799 (10.6)	.26
Nulliparous	4845 (54.9)	42 210 (45.5)	<.001
Hypertension	563 (6.4)	6147 (6.6)	.38
Pregnancy-induced hypertension	242 (2.7)	2414 (2.6)	.43
Preeclampsia	265 (3.0)	3361 (3.6)	.003
Chronic hypertension	90 (1.0)	955 (1.0)	.94
Diabetes	994 (14.0)	8475 (10.0)	<.001
Current smoking	898 (10.2)	11 104 (12.0)	<.001
Previous cesarean delivery	1199 (13.6)	17 444 (18.8)	<.001
Previous stillbirth	189 (2.1)	1156 (1.2)	<.001
EPDS score ≥12	772 (13.0)	3921 (8.8)	<.001
DFM presentations, No.			
1	7487 (84.9)	NA	NA
2	1103 (12.5)	NA	
3	231 (2.6)	NA	
Gestational age at presentation, median (IQR)	37.0 (34.0-38.5)	NA	
Planned early term birth	2028 (23.0)	18 799 (20.3)	<.001
Induction of labor	3777 (42.8)	27 205 (29.3)	<.001
Elective cesarean delivery	1395 (15.8)	18 935 (20.4)	<.001

Abbreviations: BMI, body mass index (calculated as weight in kilograms divided by height in meters squared); DFM, decreased fetal movements; EPDS, Edinburgh Postnatal Depression Scale; IQR, interquartile range.

Rates of DFM presentation, planned early term births, and stillbirth during the study period are presented in eFigure 2 in the Supplement. The number of women presenting with DFM increased by more than 4-fold from 2009 (376 of 8702 women [4.3%]) to 2019 (1733 of 9520 women [18.2%]). However, the difference in the rate of change before new national DFM guidelines were introduced vs after they were introduced was not significant, with rates changing from 376 of 8702 women (4.3%) in 2009 to 876 of 9665 women (9.1%) in 2015 and from 989 of 9490 women (10.4%) in 2016 to 1733 of 9520 women (18.2%) in 2019.

Presenting with DFM was not associated with higher odds of the primary outcome of stillbirth (9 women [0.1%] vs 185 women [0.2%]; aOR, 0.54; 95% CI, 0.23-1.26; *P* = .16). It was associated with lower odds of spontaneous vaginal birth (aOR, 0.83; 95% CI, 0.77-0.89; *P* < .001) and higher odds of planned early term birth (aOR, 1.26; 95% CI; 1.15-1.38; *P* < .001), IOL (aOR, 1.63; 95% CI, 1.53-1.74; *P* < .001), emergency cesarean delivery (aOR, 1.18; 95% CI, 1.09-1.28; *P* < .001), and an infant being born SGA (aOR, 1.14; 95% CI, 1.03-1.27; *P* = .01). Additionally, DFM was associated with higher odds of the severe perinatal outcomes composite (aOR, 1.14; 95% CI, 1.02-1.27; *P* = .01) (Table 2).

**Table 2.** Primary and Secondary Outcomes

	No. (%)		OR (95% CI)	aOR (95% CI)
	With DFM (n = 8821)	Without DFM (n = 92 776)		
Primary outcome				
Stillbirth^a^	9 (0.1)	185 (0.2)	0.51 (0.26-0.998)	0.54 (0.23-1.26)
Secondary outcomes				
Planned preterm birth^b^	171 (1.9)	2132 (2.3)	0.84 (0.72-0.98)	0.78 (0.61-1.01)
Planned early term birth^b^	2028 (23.0)	18 799 (20.3)	1.17 (1.12-1.24)	1.26 (1.15-1.38)
Induction of labor^b^	3777 (42.8)	27 205 (29.3)	1.80 (1.73-1.89)	1.63 (1.53-1.74)
Spontaneous vaginal birth^c^	4321 (49.0)	46 837 (50.5)	0.94 (0.90-0.98)	0.83 (0.77-0.89)
Emergency cesarean delivery^c^	1733 (19.6)	14 886 (16.0)	1.28 (1.21-1.35)	1.18 (1.09-1.28)
SGA^b^	854 (9.7)	7894 (8.5)	0.86 (0.80-0.93)	1.14 (1.03-1.27)
SCPO^d^	775 (8.8)	9314 (10.0)	1.15 (1.07-1.24)	1.14 (1.02-1.27)

Abbreviations: aOR, adjusted odds ratio; DFM, decreased fetal movements; OR, odds ratio; SGA, small for gestational age (ie birth weight <10th centile); SCPO, severe composite perinatal outcome (ie, neonatal critical care unit admission, severe acidosis [ie, umbilical artery pH <7.0 or base excess −12.0 mmol/L or less], 5-minute Apgar score <4, or stillbirth or neonatal death).

^a^ Adjusted for age, body mass index (calculated as weight in kilograms divided by height in meters squared), race/ethnicity, nullipara status, diabetes status, smoking status, previous cesarean delivery, previous stillbirth, Edinburgh Postpartum Depression Scale score of 12 or greater, induction of labor, elective cesarean delivery, and year of birth.

^b^ Adjusted for age, body mass index, race/ethnicity, nullipara status, diabetes status, smoking status, previous cesarean delivery, previous stillbirth, Edinburgh Postpartum Depression Scale score of 12 or greater, and year of birth.

^c^ Adjusted for age, body mass index, race/ethnicity, nullipara status, diabetes status, smoking status, previous cesarean delivery, previous stillbirth, Edinburgh Postpartum Depression Scale score of 12 or greater, induction of labor, gestational age at birth, and year of birth.

^d^ Adjusted for age, body mass index, race/ethnicity, nullipara status, diabetes status, smoking status, previous cesarean delivery, previous stillbirth, Edinburgh Postpartum Depression Scale score of 12 or greater, induction of labor, mode of birth, gestational age at birth, birth weight, and year of birth.

Additional clinical outcomes in the 2 groups are presented in Table 3. Women in the DFM group were more likely to undergo an operative vaginal birth (forceps or vacuum) or emergency cesarean delivery for presumed fetal compromise. Intrapartum pathological FHR patterns and meconium stained liquor were also more common in the DFM group. Infants in the DFM group were less likely to be born preterm (389 infants [4.4%] vs 6838 infants [7.4%]; *P* < .001) or be admitted to the NICU but more likely to have a birth weight below the fifth centile or have an Apgar score of less than 7 at 5 minutes of age. There was no difference between the 2 groups in rate of neonatal death.

**Table 3.** Additional Intrapartum and Neonatal Outcomes

Outcome	No. (%)	
	With DFM (n = 8821)	Without DFM (n = 92 776)
Intrapartum outcomes		
Vaginal birth		
Spontaneous	4321 (49.0)	46 837 (50.5)
Operative	1372 (15.6)	12 114 (13.1)
Forceps delivery	362 (4.1)	2715 (2.9)
Vacuum delivery	1010 (11.4)	9399 (10.1)
Successful induction of labor	2874 (76.1)	21 650 (79.6)
Elective cesarean delivery	1395 (15.8)	18 935 (20.4)
Emergency cesarean delivery		
For intrapartum fetal compromise	533 (6.0)	3985 (4.3)
For other reason	1200 (13.6)	10 901 (11.7)
Nonreassuring fetal status	754 (8.5)	5106 (5.5)
Meconium stained liquor	1403 (15.9)	12 426 (13.4)
Neonatal outcomes		
Gestational age at birth, median (IQR), wk	39.0 (38.0-40.0)	39.0 (38.0-40.0)
Preterm birth^a^	389 (4.4)	6838 (7.4)
Early term birth	2811 (31.9)	29 522 (31.8)
Birth weight, mean (SD), g	3388.0 (507.5)	3358.2 (551.5)
5-min Apgar score		
<7	166 (1.9)	1334 (1.4)
<3	25 (0.3)	203 (0.2)
Acidosis^b^	90 (2.0)	737 (2.1)
Respiratory distress	1243 (14.1)	13 812 (14.9)
Full term stillbirth	6 (0.1)	66 (0.1)
Gestational age of full term stillbirth, mean (SD), wk	38.7 (1.2)	38.9 (1.4)
Interval between DFM and stillbirth, median (IQR), d	28.0 (0.0-35.0)	NA
Preterm stillbirth	3 (<0.1)	119 (0.1)
Gestational age of preterm stillbirth, mean (SD), wk	35.3 (0.6)	32.2 (2.8)
Interval between DFM and stillbirth, median (IQR), d	8.0 (0.0-35.0)	NA
Neonatal death	8 (0.1)	93 (0.1)

Abbreviations: DFM, decreased fetal movements; IQR, interquartile range; NA, not applicable.

^a^ Preterm birth defined as birth earlier than 37 weeks and 0 days.

^b^ Acidosis defined as umbilical artery pH <7.0 or base excess −12.0 mmol/L or less.

Compared with women with 1 presentation for DFM, the odds of stillbirth were significantly higher in women with 2 or more presentations (aOR, 4.96; 95% CI, 0.98-24.98; *P* = .05) (Table 4) However, regardless of the number of presentations with DFM, the odds of planned early term birth were higher in women with DFM (Table 4). Neither the number of presentations with DFM nor the gestational age at which it occurred were associated with the median gestational age of stillbirth (eTable 1 in the Supplement).

**Table 4.** Primary and Secondary Outcomes by Number of DFM Presentations

	DFM						≥2 vs 1 Presentations, aOR (95% CI)
	No presentations (n = 92 776)		1 Presentation (n = 7487)		≥2 Presentations (n = 1334)		
	No. (%)	aOR	No. (%)	aOR (95% CI)	No. (%)	aOR (95% CI)	
Primary outcome							
Stillbirth^a^	185 (0.2)	1 [Reference]	6 (0.1)	0.33 (0.10-1.05)	3 (0.2)	1.62 (0.49-5.41)	4.96 (0.98-24.98)
Secondary outcomes							
Planned preterm birth^b^	2132 (2.3)	1 [Reference]	133 (1.8)	0.72 (0.54-0.96)	38 (2.8)	1.14 (0.67-1.93)	1.58 (0.88-2.84)
Planned early term birth^b^	18 799 (20.3)	1 [Reference]	1637 (21.9)	1.21 (1.10-1.33)	391 (29.3)	1.57 (1.28-1.91)	1.30 (1.05-1.61)
Induction of labor^b^	27 205 (29.3)	1 [Reference]	3079 (41.1)	1.55 (1.44-1.66)	698 (52.3)	2.25 (1.90-2.66)	1.46 (1.22-1.74)
Normal vaginal birth^c^	46 837 (50.5)	1 [Reference]	3723 (49.7)	0.85 (0.78-0.92)	598 (44.8)	0.74 (0.62-0.89)	0.88 (0.73-1.06)
Emergency cesarean delivery^c^	14 886 (16.0)	1 [Reference]	1464 (19.6)	1.19 (1.09-1.30)	269 (20.2)	1.14 (0.94-1.39)	0.96 (0.78-1.19)
Birth weight <10th centile^b^	7894 (8.5)	1 [Reference]	721 (9.6)	1.12 (1.00-1.25)	133 (10.0)	1.26 (0.99-1.61)	1.13 (0.87-1.47)
SCPO^d^	9314 (10.0)	1 [Reference]	657 (8.8)	1.15 (1.02-1.29)	118 (8.8)	1.09 (0.84-1.43)	0.95 (0.72-1.27)

Abbreviations: aOR, adjusted odds ratio; DFM, decreased fetal movements; SCPO, severe composite perinatal outcome (ie, neonatal critical care unit admission, severe acidosis [ie, umbilical artery pH <7.0 or base excess −12.0 mmol/L or lower], 5-minute Apgar score <4, or stillbirth or neonatal death).

^a^ Adjusted for age, body mass index, race/ethnicity, nullipara status, diabetes status, smoking status, previous cesarean delivery, previous stillbirth, Edinburgh Postnatal Depression Scale score of 12 or greater, induction of labor, elective cesarean delivery, and year of birth.

^b^ Adjusted for age, body mass index, race/ethnicity, nullipara status, diabetes status, smoking status, previous cesarean delivery, previous stillbirth, Edinburgh Postnatal Depression Scale score of 12 or greater, and year of birth.

^c^ Adjusted for age, body mass index, race/ethnicity, nullipara status, diabetes status, smoking status, previous cesarean delivery, previous stillbirth, Edinburgh Postnatal Depression Scale score of 12 or greater, induction of labor, gestational age at birth, and year of birth.

^d^ Adjusted for age, body mass index, race/ethnicity, nullipara status, diabetes status, smoking status, previous cesarean delivery, previous stillbirth, Edinburgh Postnatal Depression Scale score of 12 or greater, induction of labor, mode of birth, gestational age at birth, birth weight, and year of birth.

Among women with DFM, compared with women without DFM, gestational age at first presentation with DFM was not associated with odds of stillbirth. However, compared with women with DFM at later than 37 weeks, women presenting with DFM at any other gestational age had higher odds of planned early term birth, regardless of the gestational age at that presentation. The odds of vaginal birth were lower independent of gestational age at presentation with DFM, while the odds of emergency cesarean delivery were higher among women presenting with DFM before 32 weeks’ gestation or at full term compared with women without DFM. The odds of an infant being born SGA were higher among women with DFM at less than 32 weeks’ gestation compared with women without DFM. Women presenting with DFM at term had increased odds of the severe composite perinatal outcomes compared with women without DFM (eTable 2 in the Supplement).

Rates of stillbirth did not change significantly over the study period, nor did they differ between the 2 periods (pre-2016: 2 stillbirths per 1000 births vs 2016-2019: 1.8 stillbirths per 1000 births; *P* = .41). Compared with women presenting with DFM prior to 2016, women presenting with DFM after 2016 had 2-fold the odds of planned early term birth (aOR, 2.05; 95% CI, 1.71-2.45; *P* < .001) and higher odds of IOL (aOR, 1.83; 95% CI, 1.60-2.10; *P* < .001). The odds of the severe perinatal outcomes composite were lower after introduction of the national recommendations (aOR, 0.73; 95% CI, 0.8-0.91; *P* = .006) (eTable 3 in the Supplement).

## Discussion

In this cohort study at a single tertiary center, we found that DFM was not associated with higher odds of stillbirth after 28 weeks of gestation. This finding was consistent regardless of number of presentations or gestational age at first presentation with DFM. Our results are contrary to those of some other studies^4,10,14,24,32^ that found an increased risk of stillbirth among women with DFM.

However, our results suggest that risk of stillbirth may be increased among women with 2 or more presentations of DFM compared with women with 1 presentation. Consistent with results from other studies,^14,16,33,34,35^ we found that DFM was associated with higher odds of planned early term birth, IOL, and emergency cesarean delivery for intrapartum fetal compromise and lower odds of vaginal birth. Additionally, we found that women with DFM had higher rates of SGA births. Our results suggest that the association of DFM with stillbirth is likely to be mediated more by the risks associated with fetal size or perturbations in intrauterine growth rather than by DFM alone. The reasons we did not find an association between DFM and stillbirth may include a change in practice (in line with institutional guidelines and policies), heightened clinician and community awareness of the potential for adverse outcomes associated with DFM, and participation in the My Baby’s Movements’ trial.^36^ These factors resulted in higher rates of planned early term birth, which clearly resulted in increased live births. However, in our analysis of data from before and after the introduction of these guidelines, we did not find significant differences in stillbirth rates. Possible reasons for this lack of difference include changes in the demographic characteristics of women presenting with DFM and a change in the prevalence of risk factors associated with stillbirth. Interestingly, the odds of the severe perinatal outcomes composite were lower in the period after the introduction of the DFM guidelines, suggesting that earlier or planned birth may be associated with some perinatal benefit among these women.

While there are data showing that many women report a reduction in fetal movements preceding their stillbirth,^10,37^ the usefulness of DFM as a screening test for stillbirth remains unclear.^25,38,39^ Although an association between DFM and stillbirth has been found previously,^4,10,14,24,32,33^ along with other adverse perinatal outcomes,^14,16,33,34^ the difference between our study and others^14,24^ is that we excluded pregnancies for which stillbirth was diagnosed at first presentation with DFM. This was done because including such pregnancies would incorrectly influence outcomes, given that DFM is a symptom of fetal death and not a risk factor for subsequent stillbirth in these pregnancies. In our view, failure to exclude these cases from analysis in other studies may raise the potential for interpreting nonsignificant associations between DFM and stillbirth as significant. This may in part explain why stillbirth rates were not significantly different among women with DFM who were treated using strict guidelines incorporating early term birth compared with a control population in a 2018 randomized clinical trial.^25^ This trial also found that a package aimed at improving maternal awareness of DFM and expediting delivery even at early term gestation resulted in a significant increase in rates of IOL, emergency cesarean delivery, NICU admission, and early neonatal deaths.

Maternal perception of fetal movements has long been considered a sign associated with fetal well-being. Decreased fetal movements may occur as an adaptive response to fetal hypoxia (acute or chronic) as a consequence of placental dysfunction and may be associated with fetal growth restriction or stillbirth.^9^ Consequently, DFM is perceived to be an important risk factor associated with adverse perinatal outcomes. However, maternal perception of DFM is also associated with smoking, nulliparity, fetal anomaly, and anterior placental location,^9,40,41,42^ with most women who present with DFM having an uncomplicated live birth.^17^

Our finding of higher odds of planned early term birth among women with DFM is worrying but not surprising. Prevention of stillbirth is clearly achieved with the birth of a live infant, and most caregivers would feel that the trade-off between earlier birth and death is an obvious choice. However, early term birth, compared with birth at later than 39 weeks and 0 days, is associated with an increased risk of neonatal morbidity, including respiratory distress, NICU admission, neonatal hypoglycemia, and intubation.^43,44,45,46,47^ Moreover, early term birth is associated with adverse long-term neurodevelopmental outcomes, including cerebral palsy (aOR, 1.75; 95% CI, 1.32-3.31; *P* < .001) and intellectual impairment.^48^

Finally, and perhaps most importantly, we found a significant association between DFM and an infant being born SGA. In most instances, the etiology of infants born SGA is associated with suboptimal placental function.^49,50^ Indeed, there is also evidence that placentas from women with DFM are smaller with histological features associated with malperfusion.^51^ This finding supports the hypothesis that DFM may be a reflection of the fetal response to chronic hypoxia against a background of placental dysfunction and suboptimal fetal growth and fetal growth restriction. However, identification of suboptimal fetal growth on ultrasonographic images in late pregnancy is difficult, particularly for infants with estimated weights greater than the conventionally accepted threshold of the 10th centile for gestational age. There is some evidence that the fetal cerebroplacental ratio (ie, ratio of the middle cerebral artery pulsatility index to the umbilical artery pulsatility index) may be a surrogate measure of fetal growth^52,53^ with a low cerebroplacental ratio associated with an increased risk of stillbirth.^54^

Causative pathways that result in stillbirth are likely to be varied, reflecting the real-world experience that no single intervention, including public education campaigns emphasizing the importance of DFM, has effectively reduced stillbirth rates. Indeed a recent systematic review found that instructing pregnant women on fetal movement counting, compared with no instruction, did not result in improvement of pregnancy outcomes.^38,39^ There is also a lack of consensus among professional bodies regarding appropriate management of DFM.^21,22,55^ However, in Australia, there has been significant progress in the care of women at risk of stillbirth with widespread incorporation of the Safer Baby Bundle^5^ into national maternity care protocols. Although we were unable to find an association between DFM and stillbirth, we found that DFM is associated with adverse pregnancy outcomes. These results reiterate the importance of recognition of DFM as a surrogate associated with placental dysfunction and possible fetal growth restriction.

### Limitations

This study has several limitations. Our study is limited by the lack of information regarding the length of time of DFM and the elapsed duration between the start of the DFM episode and presentation, which have been found to be associated with the risk of stillbirth.^37^ Furthermore, the relatively small number of stillbirths in our study and a tertiary center focus with a clear policy algorithm for the clinical management of women with DFM may limit the generalizability of our results. Additionally, during the study period, a new clinical protocol was implemented at our center for the treatment of women with DFM. Over the study period, the absolute number of women presenting with DFM increased, but the rate of increase was similar before the introduction of national guidelines compared with after the introduction. The increase in the number of women presenting with DFM is likely associated with increased community awareness, through frequent media campaigns, of the importance of monitoring fetal movements.

## Conclusions

This cohort study found that presentation with DFM in the context of a fetus born SGA or presentation with DFM 2 or more times after 28 weeks’ gestation were associated with higher odds of stillbirth. Presentation with DFM was also a significant risk factor associated with obstetric intervention, given that it was associated with increased rates of IOL, planned early term birth, and emergency operative birth. It is arguable whether these unintended outcomes are necessarily acceptable in all health care settings given the relatively low prevalence of stillbirth late in pregnancy. The imperative for better methods of assessing fetal well-being and women’s satisfaction with planned expedited birth, as well as conducting robust health economic modeling to assess the health care costs associated with preemptive intervention, is clear. Our results suggest that any decision for obstetric intervention should not be based on the perceived risks of stillbirth solely associated with DFM and that management should be individualized, taking into account other potential associated maternal and fetal risk factors.

## Notes

Supplement. **eFigure 1.** Clinical Pathway for Management of Women Presenting With DFM After 28 Weeks’ Gestation **eFigure 2.** Time Series **eTable 1.** Median Gestational Age of Births According to No. Presentations of Decreased Fetal Movements (DFM) and Gestational Age When Presented With DFM **eTable 2.** Primary and Secondary Outcomes by Gestational Age When First Present With Decreased Fetal Movements **eTable 3.** Primary and Secondary Outcomes by Year of Birth
