# Supplementary material for: Evaluation of Pregnancy Outcomes Among Women With Decreased Fetal Movements
Source: JAMA Netw Open. 2021 Apr 8;4(4):e215071. doi: 10.1001/jamanetworkopen.2021.5071 (PMC8033440; doi:10.1001/jamanetworkopen.2021.5071)
Supplement: Supplement. — eFigure 1. Clinical Pathway for Management of Women Presenting With DFM After 28 Weeks’ Gestation eFigure 2. Time Series eTable 1. Median Gestational Age of Births According to No. Presentations of Decreased Fetal Movements (DFM) and Gestational Age When Presented With DFM eTable 2. Primary and Secondary Outcomes by Gestational Age When First Present With Decreased Fetal Movements eTable 3. Primary and Secondary Outcomes by Year of Birth [file jamanetwopen-e215071-s001.pdf]

Supplemental Online Content

Turner JM, Flenady V, Ellwood D, Coory M, Kumar S. Evaluation of pregnancy outcomes among women with decreased fetal movements. *JAMA Netw Open*. 2021;4(4):e215071. doi:10.1001/jamanetworkopen.2021.5071

eFigure 1. Clinical Pathway for Management of Women Presenting With DFM After 28 Weeks' Gestation
eFigure 2. Time Series
eTable 1. Median Gestational Age of Births According to No. Presentations of Decreased Fetal Movements (DFM) and Gestational Age When Presented With DFM
eTable 2. Primary and Secondary Outcomes by Gestational Age When First Present With Decreased Fetal Movements
eTable 3. Primary and Secondary Outcomes by Year of Birth

This supplemental material has been provided by the authors to give readers additional information about their work.

© 2021 Turner JM et al. *JAMA Network Open.*

eFigure 1. Clinical Pathway for Management of Women Presenting With DFM After 28 Weeks Gestation

At Mater Mothers' Hospital, Brisbane, implemented 2016

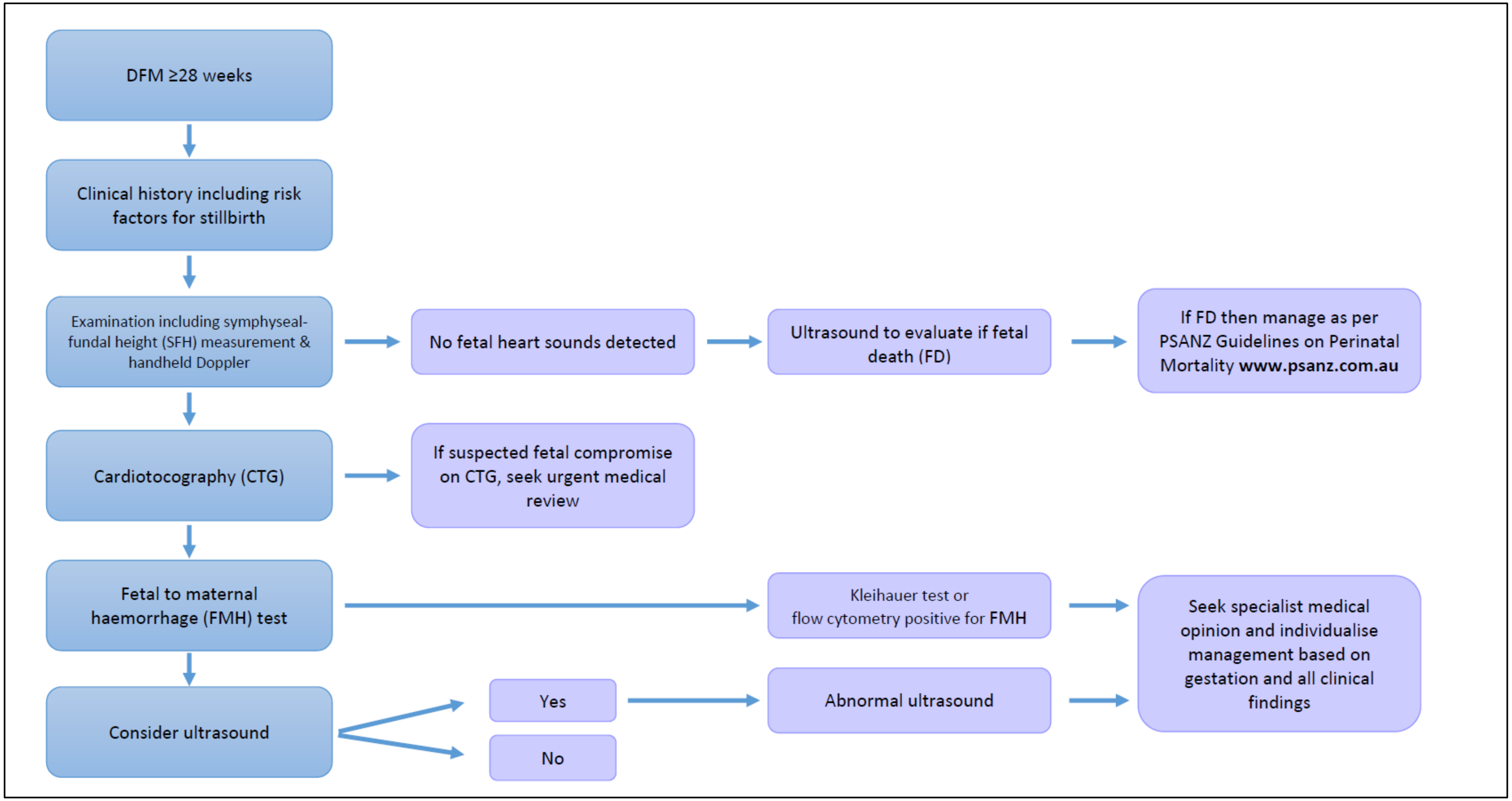

© 2021 Turner JM et al. *JAMA Network Open.*

eFigure 2. Time Series

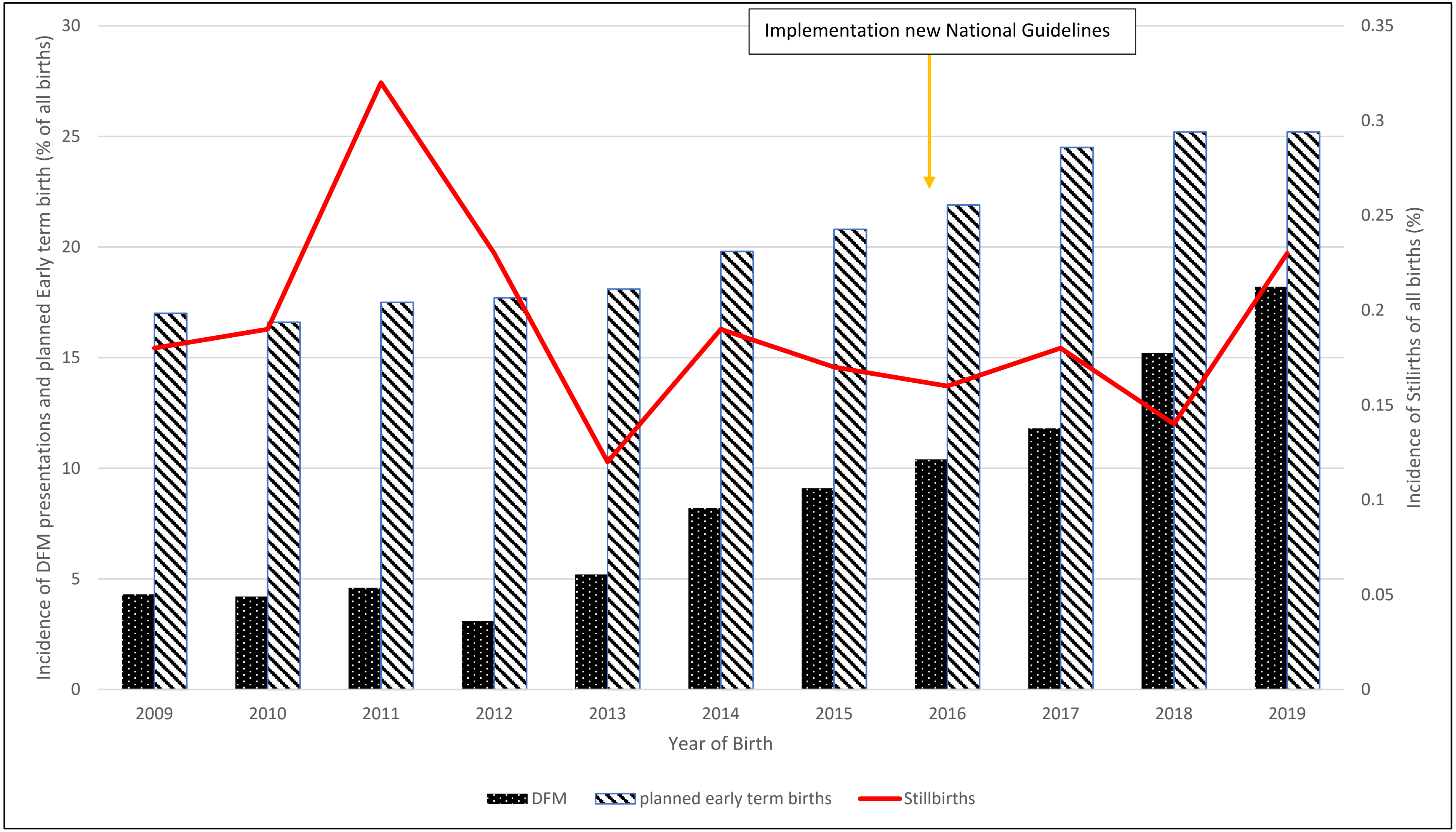

Illustration of rates of decreased fetal movement (DFM) presentations, planned early term births and stillbirth during study period

© 2021 Turner JM et al. *JAMA Network Open.*

eTable 1. Median Gestational Age of Births According to No. Presentations of Decreased Fetal Movements (DFM) and Gestational Age When Presented With DFM

	Live Birth	P value	Preterm Stillbirth	P value	Term Stillbirth	P value
**No DFM**	39 (38-40)		32 (30-35)		39 (38-40)	
**1 presentation DFM**	39 (38-40)	0.0001	35.5 (35-36)	0.14	39 (37.5-40)	0.92
**≥2 presentation DFM**	39 (38-40)		35 (35-35)		38.5 (38-39)	
**No DFM**	39 (38-40)		32 (30-35)		39 (38-40)	
**DFM 28-31+6 weeks**	39 (38-40)		36 (36-36)		-	
**DFM 32-36+6 weeks**	38 (38-39)	0.0001	35 (35-35)	0.13	38 (38-40)	0.94
**DFM ≥37 weeks**	40 (39-40)		-		39 (39-39)	

Data presented as median (IQR); DFM, decreased fetal movements

eTable 2. Primary and Secondary Outcomes by Gestational Age When First Present With Decreased Fetal Movements

	**No DFM**		**DFM at 28-32 weeks**		**DFM at 32-36+6weeks**		**DFM at >=37 weeks**	
	**N=92,776**		**N=1,736**		**N=3,088**		**N=3,997**	
	**% (n)**	**aOR**	**% (n)**	**aOR (95% CI)**	**% (n)**	**aOR (95% CI)**	**% (n)**	**aOR (95% CI)**
**PRIMARY OUTCOME**								
**Stillbirth***	185 (0.2%)	1	1 (0.06%)	1	7 (0.2%)	1.38 (0.54-3.51)	1 (0.0%)	0.18 (0.03-1.33)
**SECONDARY OUTCOMES**								
**Planned preterm birth$**	2,132 (2.3%)	1	76 (4.4%)	1.66 (1.12-2.47)	94 (3.0%)	1.37 (0.98-1.90)	1 (0.03%)	1
**Planned early term birth$**	18,799 (20.3%)	1	500 (28.8%)	1.75 (1.46-2.09)	987 (32.0%)	1.85 (1.62-2.12)	541 (13.5%)	0.72 (0.62-0.84)
**Induction of Labour$**	27,205 (29.3%)	1	731 (42.1%)	1.54 (1.32-1.79)	1,300 (42.1%)	1.69 (1.51-1.89)	1,746 (43.7%)	1.63 (1.49-1.79)
**Normal Vaginal Birth#**	46,837 (50.5%)	1	761 (43.8%)	0.75 (0.63-0.88)	1,413 (45.8%)	0.84 (0.74-0.96)	2,147 (53.7%)	0.86 (0.78-0.94)
**Emergency CS#**	14,886 (16.0%)	1	374 (21.5%)	1.19 (1.00-1.42)	593 (19.2%)	1.07 (0.93-1.23)	766 (19.2%)	1.26 (1.13-1.41)
**Birthweight <10th centile$**	7,894 (8.5%)	1	185 (10.7%)	1.32 (1.06-1.65)	294 (9.5%)	1.16 (0.97-1.38)	375 (9.4%)	1.07 (0.93-1.23)
**SCPO%**	9,314 (10.0%)	1	200 (11.5%)	1.15 (0.93-1.43)	301 (9.7%)	1.01 (0.85-1.20)	274 (6.9%)	1.25 (1.07-1.48)

DFM, decreased fetal movements; OR, odds ratio; aOR, adjusted odds ratio; 95% CI, 95% confidence interval; Perinatal death, stillbirth or neonatal death; CS, caesarean section; SCPO, severe composite perinatal outcome [either neonatal critical care unit admission, acidosis (cord pH ≤7.0 or base excess ≤-12.0), 5-minute Apgar score <3 or perinatal death]]; EDS, Edinburgh depression score

[a] *adjusted for age, BMI, ethnicity, nullipara, diabetes, smoker, previous CS, previous stillbirth, EDS ≥12, induction of labour, elective CS and year of birth*

[b] *adjusted for age, BMI, ethnicity, nullipara, diabetes, smoker, previous CS, previous stillbirth, EDS ≥12 and year of birth*

[c] *adjusted for age, BMI, ethnicity, nullipara, diabetes, smoker, previous CS, previous stillbirth, EDS ≥12, induction of labour, gestation at birth and year of birth*

[d] *adjusted for age, BMI, ethnicity, nullipara, diabetes, smoker, previous CS, previous stillbirth, EDS ≥12, induction of labour, mode of birth, gestation at birth, birthweight and year of birth*

© 2021 Turner JM et al. *JAMA Network Open.*

eTable 3. Primary and Secondary Outcomes by Year of Birth

	2009-2015			2016-2019			DFM pre-2016 vs DFM post-2016
	DFM	No DFM		DFM	No DFM		
	N=3,530	N=60,048		N=5,291	N=32,728		
	N (%)	N (%)	aOR (95% CI)	N (%)	N (%)	aOR (95% CI)	aOR (95% CI)
**PRIMARY OUTCOME**							
**Stillbirth** [a]	2 (0.06%)	125 (0.2%)	0.32 (0.08-1.32)	7 (0.1%)	60 (0.2%)	1.03 (0.34-3.16)	1.70 (0.33-8.76)
**SECONDARY OUTCOMES**							
**Planned preterm birth**[b]	63 (1.8%)	1,376 (2.3%)	0.76 (0.54-1.07)	108 (2.0%)	756 (2.3%)	0.84 (0.57-1.23)	1.05 (0.62-1.75)
**Planned early term birth**[b]	570 (16.1%)	11,043 (18.4%)	1.17 (1.02-1.34)	1,458 (27.6%)	7,756 (23.7%)	1.42 (1.27-1.60)	2.05 (1.71-2.45)
**Induction of labour**[b]	1,288 (36.5%)	16,298 (27.1%)	1.53 (1.40-1.68)	2,489 (47.0%)	10,907 (33.3%)	1.87 (1.70-2.07)	1.83 (1.60-2.10)
**Normal vaginal birth**[c]	1,800 (51.0%)	30,650 (51.0%)	0.86 (0.78-0.95)	2,521 (47.6%)	16,187 (49.5%)	0.80 (0.71-0.89)	0.91 (0.79-1.06)
**Emergency CS**[c]	680 (19.3%)	9,424 (15.7%)	1.15 (1.03-1.29)	1,053 (19.9%)	5,462 (16.7%)	1.17 (1.04-1.32)	1.01 (0.85-1.19)
**Birthweight <10th centile**[b]	361 (10.2%)	5,035 (8.4%)	1.22 (1.06-1.40)	493 (9.3%)	2,859 (8.7%)	1.05 (0.90-1.23)	0.88 (0.71-1.10)
**SCPO**[d]	357 (10.1%)	6,532 (10.9%)	1.11 (0.96-1.28)	418 (7.9%)	2,782 (8.5%)	1.14 (0.96-1.35)	0.73 (0.58-0.91)

DFM, decreased fetal movements; OR, odds ratio; aOR, adjusted odds ratio; 95% CI, 95% confidence interval; Perinatal death, stillbirth or neonatal death; CS, caesarean section; SCPO, severe composite perinatal outcome [either neonatal critical care unit admission, acidosis (cord pH ≤7.0 or base excess ≤-12.0), 5-minute Apgar score <3 or perinatal death]]; EDS, Edinburgh depression score

[a] *adjusted for age, BMI, ethnicity, nullipara, diabetes, smoker, previous CS, previous stillbirth, EDS ≥12, induction of labour and elective CS*

[b] *adjusted for age, BMI, ethnicity, nullipara, diabetes, smoker, previous CS, previous stillbirth and EDS ≥12*

[c] *adjusted for age, BMI, ethnicity, nullipara, diabetes, smoker, previous CS, previous stillbirth, EDS ≥12, induction of labour and gestation at birth*

[d] *adjusted for age, BMI, ethnicity, nullipara, diabetes, smoker, previous CS, previous stillbirth, EDS ≥12, induction of labour, mode of birth, gestation at birth and birthweight*

© 2021 Turner JM et al. *JAMA Network Open.*
